# Utility of the Current Procedural Terminology Codes for Prophylactic Stabilization for Defining Metastatic Femur Disease

**DOI:** 10.5435/JAAOSGlobal-D-20-00167

**Published:** 2020-12-18

**Authors:** Sarah M. Hanna, Duncan C. Ramsey, Yee C. Doung, James B. Hayden, Reid F. Thompson, Andrew R. Summers, Kenneth R. Gundle

**Affiliations:** From the Department of Orthopaedics & Rehabilitation, Oregon Health & Science University, Portland, OR (Ms. Hanna, Dr. Ramsey, Dr. Doung, Hayden, Dr. Gundle); the Portland VA Medical Center Operative Care Division (Dr. Ramsey, Dr. Gundle) and the Division of Hospital and Specialty Medicine (Dr. Thompson), Portland, OR; the Department of Radiation Medicine, Oregon Health & Science University (Dr. Thompson), Portland, OR; and the Department of Orthopaedic Surgery, University of Pennsylvania (Dr. Summers), Philadelphia, PA.

## Abstract

**Introduction:**

Cohorts from the electronic health record are often defined by the Current Procedural Terminology (CPT) codes. The error prevalence of CPT codes for patients receiving surgical treatment of metastatic disease of the femur has not been investigated, and the predictive value of coding ontologies to identify patients with metastatic disease of the femur has not been adequately discussed.

**Methods:**

All surgical cases at a single academic tertiary institution from 2010 through 2015 involving prophylactic stabilization of the femur or fixation of a pathologic fracture of the femur were identified using the CPT and International Classification of Disease (ICD) codes. A detailed chart review was conducted to determine the procedure performed as documented in the surgical note and the patient diagnosis as documented in the pathology report, surgical note, and/or office visit notes.

**Results:**

We identified 7 CPT code errors of 171 prophylactic operations (4.1%) and one error of 71 pathologic fracture fixation s(1.4%). Of the 164 prophylactic operations that were coded correctly, 87 (53.0%) had metastatic disease. Of the 70 pathologic operations that were coded correctly, 41 (58%) had metastatic disease.

**Discussion:**

The error prevalence was low in both prophylactic stabilization and pathologic fixation groups (4.1% and 1%, respectively). The structured data (CPT and ICD-9 codes) had a positive predictive value for patients having metastatic disease of 53% for patients in the prophylactic stabilization group and 58% for patients in the pathologic fixation group. The CPT codes and ICD codes assessed in this analysis do provide a useful tool for defining a population in which a moderate proportion of individuals have metastatic disease in the femur at an academic medical center. However, verification is necessary.

Anticipated benefits of the electronic health record (EHR) included the ability to use existing patient data to rapidly answer research questions and provide accurate quality metrics. Exposures and outcomes based on EHR data are often defined by administrative code data, such as the Current Procedural Terminology (CPT) codes and International Classification of Disease (ICD) codes. However, even with multiple available coding systems, identifying patients in a target cohort can be challenging. Difficulties can arise from the fact that administrative data are not collected for the purpose of defining research cohorts. Data integrity can further suffer from inaccuracy, incompleteness, and a lack of standardization across multiple providers and institutions.^1,2^

Previous research on the use of EHR data to identify patients of interest has largely focused on tracking the outcomes of surveillance significance, such as healthcare-associated infections.^3,4,5,6^ Although some studies have investigated the use of EHR data to identify patients with rare diseases, these studies have generally not included an analysis of the error prevalence of CPT coding, and thus far no studies have been published regarding the use of structured data to identify patients with bony metastases.^7-9^

Metastatic bone disease is relatively common, affecting an estimated 280,000 to 330,000 people in the United States.^10,11^ However, most studies on metastatic bone disease are small, single-center studies^12,13,14,15,16^ or use larger databases with limitations that stem from retrospective design, low granularity, and lack of statistical control.^17^ Some studies have used the CPT and ICD codes to define cohorts of patients with metastatic disease of the femur,^17^ whereas others do not explicitly state the criteria or methodology that were used to identify patient cohorts.^12,13,14,15,16,18-20^ Efforts to produce studies with larger sample sizes will likely rely on structured data to identify patient cohorts. A lack of understanding of the characteristics and accuracy of cohorts defined by ICD and CPT codes could lead to biased conclusions.

The CPT and ICD ontologies are candidates to identify patients requiring surgical treatment of metastatic disease of the femur. This study aimed to characterize the error prevalence and utility of the CPT and ICD codes in the identification of two cohorts of interest: patients who received prophylactic stabilization of the femur for metastatic disease and patients who underwent surgical fixation of completed pathologic femur fractures because of metastatic bone disease.

## Methods

### Data Source and Study Design

This is a cross-sectional study at a single academic medical center. The study design and methods for data collection were approved by the Institutional Review Board.

### Study Sample

All surgical cases at a single academic tertiary institution from 2010 through 2015 involving prophylactic stabilization of the femur or fixation of a pathologic fracture of the femur were identified using the CPT and ICD codes as outlined in Table 1. The selected time frame was used to assess ICD-9 codes without the influence of the ICD-10 transition. Briefly, prophylactic stabilization cases were identified using CPT codes 27495 or 27187. Pathologic fixation cases were identified using CPT codes 27236, 27244, 27245, 27269, 27506, or 27511 plus ICD-9 codes 733.10, 733.14, or 733.15. A total of 171 prophylactic stabilization and 71 pathologic fracture cases were identified.

**Table 1:** CPT and ICD Codes Used to Query Patients From the Database

For Prophylactic Stabilization Cases: Any of the CPT Codes Below	For Pathologic Fixation Cases: Any of the CPT Codes Below Combined With Any of the ICD Codes Below	
CPT Codes	CPT Codes	ICD Codes
27495: prophylactic treatment (nailing, pinning, plating, or wiring) with or without methylmethacrylate and femur	27236: open treatment of femoral fracture, proximal end, neck, internal fixation, or prosthetic replacement	733.14: pathologic fracture neck of femur
27187: prophylactic treatment (nailing, pinning, plating, or wiring) with or without methylmethacrylate, femoral neck, and proximal femur	27244: treatment of intertrochanteric, peritrochanteric, or subtrochanteric femoral fracture, with plate/screw type implant, with or without cerclage	733.15: pathologic fracture other part of femur
	27245: treatment of intertrochanteric, peritrochanteric, or subtrochanteric femoral fracture, with intramedullary implant, with or without interlocking screws and/or cerclage	733.10: pathologic fracture unspecified site
	27269: open treatment of femoral fracture, proximal end, and head, includes internal fixation, when performed	
	27506: open treatment of femoral shaft fracture, with or without external fixation, with insertion of intramedullary implant, with or without cerclage and/or locking screws	
	27511: open treatment of femoral supracondylar or transcondylar fracture without intercondylar extension, includes internal fixation, when performed	

CPT = Current Procedural Terminology, ICD = International Classification of Disease

### Study Variables

Patient date of birth, age at the time of surgery, sex, CPT codes, and ICD codes were abstracted as part of the database query. Manual chart review was conducted to determine body mass index, race, the American Society of Anesthesiologists score as documented in the anesthesia note, the procedure performed, and whether the patient had a diagnosis of metastatic cancer. There were two main outcome variables of interest. The first was the proportion in which the procedure outlined in the surgicaloperative note matched the CPT code. The second was the proportion in which the pathology report, surgical note, or office visit notes documented a diagnosis of metastatic cancer or myeloma in patients who were identified as such by the corresponding CPT and ICD codes as defined above.

### Statistics

Proportions of patients with correct CPT codes are reported. Of patients with correct CPT codes, the proportion of patients who had metastatic disease on chart review are also reported. Correlations are analyzed using a two-sided chi-square tests or Fisher exact test. Analyses were preplanned, and an alpha <0.05 was taken as significant. All statistics were conducted in R version 3.6.1.^21^

## Results

Patient characteristics are shown in Table 2. The mean patient age in both the prophylactic and pathologic groups was approximately 55 years. The prophylactic group had a slight majority of women (54%), whereas in the pathologic group, women were a slight minority (48%). Patients in both groups were overwhelmingly Caucasian, which is consistent with the demographics of the area served by the study institution.

**Table 2:** Patient Characteristics by Procedure Type

	Prophylactic Stabilization (n = 171)	Pathologic Fixation (n = 71)
Age, mean (SD)	55.17 (19.69)	55.19 (21.97)
Sex, n (%)		
Male	78 (46)	37 (52)
Female	93 (54)	34 (48)
BMI category n (%)		
Underweight	4 (2)	5 (7)
Normal weight	61 (36)	27 (38)
Overweight	38 (22)	20 (28)
Class I obesity	40 (23)	11 (16)
Class II obesity	15 (9)	4 (6)
Class III obesity	13 (8)	4 (6)
Race and ethnicity, n (%)		
Caucasian	148 (87)	61 (86)
Black	4 (2)	0 (0)
Hispanic	6 (4)	5 (7)
Asian	6 (4)	1 (1)
American Indian or Alaskan Native	2 (1)	0 (0)
Native Hawaiian or Pacific Islander	0 (0)	1 (1)
Other/multiracial	3 (2)	2 (3)
ASA, n (%)		
1	14 (8)	5 (7)
2	57 (33)	20 (28)
3	84 (49)	38 (54)
4	14 (8)	7 (10)

ASA = American Society of Anesthesiologists, BMI = body mass index

After comparison to the surgical reports, we identified seven CPT code errors of 171 (4.1%) prophylactic operations and one error of 71 (1.4%) pathologic fracture fixations (Table 3). Among cases incorrectly coded as prophylactic stabilization, three cases incorrectly applied a CPT of 27187 and four cases incorrectly applied a code of 27495. The incorrectly applied 27187 codes were applied to one case of hemiarthroplasty, one case of revision of hardware, and one case of a documented fracture. The incorrectly applied 27495 codes were applied to two cases of revisions of stabilization or fixation of osteotomies, one case of removal of an antibiotic spacer, and one case of an explant of a total knee arthroplasty (Table 4). For the single miscoded pathologic fixation case, a CPT code of 27506 was incorrectly applied for removal and replacement of screws in an existing femoral intramedullary nail.

**Table 3:** CPT Code Error Counts by Procedure Type

	Total	Correct CPT	Incorrect CPT
Prophylactic stabilization, n (%)	171	164 (96)	7 (4)
Pathologic fixation, n (%)	71	70 (99)	1 (1)

CPT = Current Procedural Terminology

CPT errors were not associated with procedure type (two-sided Fisher exact test, *P* = 0.44).

**Table 4:** List of All Miscoded Procedures by Procedure Type

Prophylactic Cases			Pathologic Cases		
Prophylactic Miscode Case	CPT Code	Reason-Deemed Miscode	Pathologic Miscode Case	CPT Code	Reason-Deemed Miscode
1	27187^a^	Operation was right hip hemiarthroplasty	1	27506^b^	Procedure was removing and replacing screws in existing femoral intramedullary nail
2	27187^a^	Operation was revision of hardware of the left proximal femur			
3	27187^a^	Patient had a fracture documented by the surgeon; treatment was not prophylactic			
4	27495^c^	Operation was exchange of antibiotic spacer, right distal femur arthroplasty			
5	27495^c^	Operation was revision of stabilization of left femur osteotomy			
6	27495^c^	Operation was revision of fixation right femoral osteotomies with plates and screws			
7	27495^c^	Operation was explant of right total knee arthroplasty			

CPT = Current Procedural Terminology

a Prophylactic treatment (nailing, pinning, plating, or wiring) with or without methyl methacrylate, femoral neck, and proximal femur.

b Open treatment of femoral shaft fracture, with or without external fixation, with insertion of intramedullary implant, with or without cerclage and/or locking screws.

c Prophylactic treatment (nailing, pinning, plating, or wiring) with or without methy lmethacrylate, femur.

The CPT code that was applied and the reason the case was considered a miscode are shown.

Of the 164 prophylactic operations that were coded correctly, 87 (53.0%) had metastatic disease as verified by manual chart review (Table 5). Of the 70 pathologic operations that were coded correctly, 41 (58%) had metastatic disease as verified by manual chart review (Table 5). Thus, the CPT and ICD codes had a positive predictive value for patients having metastatic disease as determined by chart review of 53% for patients in the prophylactic stabilization group and 58% for patients in the pathologic fixation group. The proportion of patients with metastatic disease in the group of patients with correct CPT codes did not differ significantly between the pathologic fixation and prophylactic stabilization groups (χ^2^ = 0.69789, df = 1, *P* = 0.4035). In both the prophylactic stabilization and pathologic fixation cohorts with metastatic bone disease, the most common diagnosis was metastatic carcinoma (77% and 71%, respectively), followed by metastatic hematologic malignancy (18% and 22%, respectively) (Table 6). In both the prophylactic stabilization and pathologic fixation cohorts without metastatic bone disease, the most common diagnosis was a benign lesion (69% and 52%, respectively). The second most common diagnosis for prophylactic stabilization patients without metastatic disease was soft-tissue sarcoma (25%); these patients underwent prophylactic stabilization because of the use of radiation and periosteal stripping during their operations. For pathologic fixation patients without metastatic disease, the next most common diagnoses were soft-tissue sarcoma and primary bone cancer (14% each). For the primary bone tumors, in addition to resection, these patients underwent reconstructions which met the inclusion criteria by used CPT codes.

**Table 5:** TheNumber of Patients With Correct CPT Codes Who Had Metastatic Disease of the Femur on Chart Review Versus Did Not Have Metastatic Disease of the Femur on Chart Review by Procedure Type (χ^2^ = 0.69789, df = 1, *P* = 0.4035)

	Total	Presence of Metastatic Disease to the Femur on Chart Review	Absence of Metastatic Disease to the Femur on Chart Review
Prophylactic stabilization, n (%)	164	87 (53)	77 (47)
Pathologic fixation, n (%)	70	41 (58)	28 (42)

CPT = Current Procedural Terminology

**Table 6:** Types of Lesions for Patients With Metastatic Disease to the Femur (Top) and Without Metastatic Disease of the Femur (Bottom) by Surgery Type

	Prophylactic Stabilization	Pathologic Fixation
Presence of metastatic disease to the femur on chart review	87	41
Metastasis from carcinoma	67 (77)	29 (71)
Metastasis from hematologic cancer	16 (18)	9 (22)
Metastasis from melanoma	2 (2)	3 (7)
Metastasis from primary bone tumor	2 (2)	0 (0)
Absence of metastatic disease to the femur on chart review	77	29
Benign lesion	53 (69)	15 (52)
Aneurysmal bone cyst	3	3
Brown tumor	2	0
Chondroblastoma	3	0
Low-grade chondroid neoplasm	8	0
Desmoplastic fibroma	1	0
Fibrous dysplasia	12	2
Giant cell tumor	4	0
Osteochondroma	4	1
Pigmented villonodular synovitis	1	0
Unicameral bone cyst	8	1
Nonneoplastic, no specific pathologic diagnosis	7	8
Infection	1 (1)	2 (7)
Nonmetastatic primary bone cancer	0 (0)	4 (14)
Soft-tissue sarcoma	20 (26)	4 (14)
Radiation-induced lesion	0 (0)	1 (3)
Other	3 (4)	3 (10)

Percentages that do not add to 100 are because of rounding error.

Data are presented as n (%).

Diagnoses for patients who did not have metastatic disease included severe osteoporosis, previously radiated soft-tissue sarcomas, benign bone lesions, and metabolic diseases affecting the bone.

## Discussion

### Key Findings

Over the study period, the data suggest there was a CPT code error prevalence of 4% for patients in the prophylactic stabilization group and 1% in the pathological fracture group, although these were not necessarily for *metastatic* disease. This low error prevalence is likely related to the importance of accurate coding at academic institutions for appropriate billing.

The structured data used (CPT and ICD-9 codes) had a positive predictive value for patients having metastatic disease of 53% for patients in the prophylactic stabilization group and 58% for patients in the pathologic fixation group. This low positive predictive value may be due to an unusually high number of patients with soft-tissue sarcomas, primary bone lesions, and other unusual surgical diagnoses (including osteogenesis imperfecta and severe osteoporosis for example) at this tertiary academic center. The positive predictive value may be higher at other institutions.

### Strengths and Limitations

The strength of this study design was the use of manual chart review for several key unstructured fields. Review of individual surgical notes, pathology notes, and office visit notes was critical to determine the primary and secondary outcomes in this study. In contrast to many previous studies, an estimate of CPT coding error prevalence is presented. It is important to establish coding error prevalence because a high error rate would certainly confound attempts to accurately identify a cohort of patients with metastatic disease to the femur using administrative code data.

Because the study cohorts were defined a priori using the CPT and ICD codes, not all patients receiving prophylactic stabilization or pathologic fixation for metastatic disease of the femur were necessarily captured, given the inherent CPT error prevalence for this method described in this study. A manual chart review of all femoral stabilization and arthroplasty procedures to confirm whether a pathologic fracture existed is rarely practical. We can therefore not speak to the true sensitivity, specificity, or negative predictive value of this ontology.

In addition, the predictive value of the ICD and CPT codes may have changed with the transition to ICD-10 codes. However, many database studies have been performed or are still performed using data before the transition, and these data provide a useful lens through which to view these studies.

Finally, this study is limited by the use of a single academic medical center with a relatively small group of patients. These CPT codes may better identify patients with metastases to the femur in the community setting, where there are likely to be fewer patients receiving surgical treatment of rare metabolic diseases or radiation-related fractures from previous soft-tissue sarcomas.

### Significance

The ability to quickly define cohorts, exposures, and outcomes based on data contained in medical charts can accelerate the pace of discovery in biomedical and public health research and provide more meaningful and reliable quality metrics.^2-4^ However, this depends on accurately identifying cohorts of interest. Our results suggest that the CPT codes for prophylactic femur stabilization and fixation of pathologic femur fractures are rarely miscoded but are inadequate to define a cohort of patients with metastatic disease. This suggests a major limitation in the use of administrative databases for research on this patient population without additional means of identifying metastatic disease. The wide variety of primary ICD codes associated with the procedures also limits the utility of simply combining the CPT with ICD codes.

Our findings are most directly relevant to database studies and other studies using CPT and ICD codes to identify patients without verification by chart review.^22^ Such use of administrative databases for orthopaedic clinical research is rising steadily.^23,24^ Our data suggest identification of patients requiring surgical treatment of metastatic bone disease of the femur using structured data alone is likely to produce biased results. The cohorts we identified in this way contained a high proportion of patients without metastatic disease. In the study by Phillipp et al, for example, we expect their estimates of survival are probably overestimates.^17^

These findings also highlight the importance of explicitly stating the process by which patients are identified in retrospective cohort studies for metastatic bone disease, considering that CPT coding errors would lead to the inclusion of patients outside a given target cohort. In several of the small cohort studies on metastatic bone disease of the femur we reviewed, the methods suggested that a chart review for data abstraction took place, but it was unclear if or how the chart review contributed to the definition of eligible patients.^12,13,14,15,16,18-20,25^

The CPT codes assessed in this analysis do provide a useful tool for defining a population in which a moderate proportion of individuals have metastatic disease in the femur at an academic medical center. However, verification is necessary, and individual verification by chart review is time consuming. Algorithms based on structured and unstructured EHR data may facilitate this process, although even algorithms with good sensitivity and specificity have poor positive predictive value for rare diseases.^6,9^ Our results suggest that structured data may be useful to screen in a population of individuals with a higher prevalence of a rare disease, which would allow algorithms based on further structured and unstructured data to better identify patients in the cohort of interest. Because most our diagnosis verification was performed via review of surgical and pathology reports, we propose that a patient identification algorithm based on natural language processing may facilitate verification.
